# Supplementary material for: Identification of Freezing-Responsive microRNAs and Their Targets in Chinese Jujube by Small RNA and Degradome Sequencing
Source: Int J Mol Sci. 2026 Sep 4;27(17):7912. doi: 10.3390/ijms27177912 (PMC13566890; doi:10.3390/ijms27177912)
Supplement: Supplementary file 1 [file ijms-27-07912-s001.zip › Supplementary Material S2/novel_miR_148.pdf]

Provisional ID : novel_miR_148
Score total : 0.4
Score for star read(s) : -1.3
Score for read counts : 0
Score for mfe : 0.1
Score for randfold : 1.6
Score for cons. seed :
Total read count : 57
Mature read count : 57
Loop read count : 0
Star read count : 0

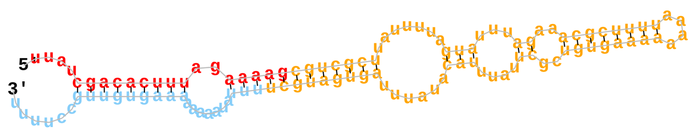

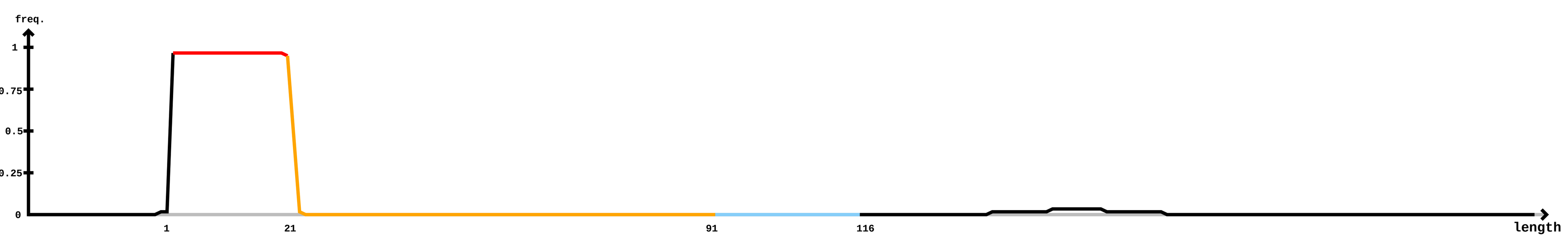

Mature Star

```
5'- gaaaaguauguuauccuaauauuuaucgacacuuuagaaaagcgucgcuuauuuuaguauuuagaaacgcuuuuaaaaaaaagugucgcuuauuuacauauuuagugaugcuuuuauaaaaaaaaaguguugccuuuuauuuuuauuuuauuuuuccuuccaauuugaaaguugcuuuuaguuuucucaaauuaugacuguuuaauaguuaaauucauguaaaauaauuaauagacaacaacguucaaaua -3'   exp
    ((((((((...((....((((.....((((((((..(((((((((((.......(((...((..((((((((....))))))))..))....)))......))))))))))).......))))))))).....))))..))...))))))))......((((((.((((((.((((((....((((((.(((((((...)))))))))))..))......)))))).))....)))).))))))).   reads   mm   sample
    ....................auuuaucgacacuuuagaaGagcg...............................................................................................................................................................................   1   1   LT-3
    ......................uuaucgacacuuuagaaaU..................................................................................................................................................................................   1   1   LT-3
    ......................uuaucgacacuuuagaaaag.................................................................................................................................................................................   26   0   LCK-1
    ......................uuaucgacacuuuNgaaaag.................................................................................................................................................................................   1   1   LCK-1
    ......................uuaucgacacuuuagaaaag.................................................................................................................................................................................   27   0   LCK-2
    ......................uuaucgCcacuuuagaaaag.................................................................................................................................................................................   1   1   LCK-2
    ..............................................................................................................................................................ccGauuugaaaguugcuuu..........................................................................   1   1   LT-3
    ........................................................................................................................................................................aguugcuuGuaguuuucuc................................................................   1   1   LCK-1
```
